# ‘It Makes Me Feel Old’: Understanding the Experience of Recovery From Ankle Fracture at 6 Months in People Aged 50 Years and Over

**DOI:** 10.1177/10497323231153605

**Published:** 2023-02-06

**Authors:** Elizabeth Tutton, Jenny Gould, Sarah E. Lamb, Matthew L. Costa, David J. Keene

**Affiliations:** 1 Nuffield Department of Orthopaedics, Rheumatology and Musculoskeletal Sciences, 6396 University of Oxford, Oxford, UK; 2 Major Trauma Centre, 6396 Oxford UniversityHospitals NHS Foundation Trust, Oxford, UK; 3 Faculty of Health and Life Sciences, 3286 University of Exeter, Exeter, UK

**Keywords:** qualitative, interviews, older people, research, ankle fracture, experience

## Abstract

Ankle fracture is a common injury, and depending on injury severity, treatment may be a support boot, cast or surgery. Older people, particularly those with severe injuries who are asked to restrict weight bearing, struggle with early recovery. To elicit older peoples’ experience of recovery 6 months after injury, we drew on a phenomenological approach using interviews. Findings revealed that getting on with life was a way of accepting what it feels like to ‘be vulnerable’, needing to ‘be safe’ while determinedly working hard to ‘be myself’. Being vulnerable identified endurance of inactivity, loneliness and dependency in the non-weight bearing period of recovery, followed by a struggle to weight bear while lacking confidence and being fearful of falling and causing further damage. Being safe conveyed fragility where sensations, pain and stiffness acted as bodily reminders of injury. Lack of function and awareness of danger led to carefulness where planning or curtailing of activities ensured their safety. Being myself showed a determination to push away from a disrupted self-identify of being older or disabled while being challenged by the continuous process of learning to be more mobile. A lack of readiness for old age created a drive to age well. Despite loss of ability, participants hoped to regain their pre-injury way of living. This study challenges practice that disregards the hard work required to recover from ankle fracture. As comorbidity increases with age, failure to consider this aspect may contribute to frailty in this group of older people.

## Introduction

Ankle fracture is a common injury constituting 9% of all fractures in adults (Court-Brown & Caesar, 2006). People aged 50 years and over have a higher incidence of ankle fracture (Curtis et al., 2016), and this is commonly a fall from standing, referred to as a fragility fracture (Kannus et al., 2008). Ankle fracture treatment varies according to injury severity, such as a cast or boot for less severe and surgery or close contacting casting for more severe injuries. In the latter, a reduction in function and walking ability at 6 months after ankle fracture and a wide variation in physiotherapy provision following treatment was found (Willett et al., 2016). In order to examine best practice for rehabilitation in older people with an ankle fracture, a randomised feasibility study exploring different rehabilitation approaches for adults aged 50 years and over was undertaken. This qualitative study was situated within this larger feasibility study (Keene et al., 2019; Keene et al., 2022).

The incidence of ankle injury in older people is likely to increase (Kannus et al., 2008), and a detailed understanding of the experience of recovery at key time points is required to inform interventions to enhance recovery. Qualitative research has identified the struggle to live with an ankle fracture in early recovery and that ongoing symptoms and changes in daily life continue up to 2 years post injury. Older people (>60 years) with more severe injury at 6–10 weeks identified the challenge of coping with non-weight bearing (Keene et al., 2016). Participants’ endured pain and renegotiated roles and relationships as dependency disrupted their daily life. Inventive ways to achieve simple tasks and ways to keep busy were found. In their struggle to move, they coped with hopping and co-morbidities and were challenged by walking aids. Getting back to walking and bearing weight on their injured leg was hard and they worried about long-term recovery. Other studies with adults support this evidence with the addition of a lack of information about the pathway to recovery (Jensen et al., 2021) and concerns about sleep and fatigue (McKeown et al., 2020). Similarly, adults up to 2 years after injury continue to identify discomfort, swelling, reduced function, low mood, reduced income and challenges in their ability to work (McPhail et al., 2012).

These studies provide the groundwork for understanding the broad experience of recovery from ankle fracture, although description of methodology is often limited. There remains a gap in our detailed understanding of recovery from ankle fracture at specific time points. All ankle fractures impose restrictions on mobility. People with more severe injuries, whether treated surgically or non-surgically, where weight bearing is restricted, often struggle to recover. We therefore need to know more about the lived experience of this group. Currently, there is limited understanding of older peoples’ experience of recovery from ankle fracture at 6 months. This study therefore explores the research question, what is the experience of recovery from ankle fracture in older people, at 6 months after injury?

## Methods

The methodology was informed by Heideggerian Phenomenology that considers what it is like to be in the world through the notion of Dasein (being or presence) (Heidegger, 1962). Through questioning, access can be gained into the experience of others ‘understandings, feelings and perceived relationships’ of everyday life (p3) (Van Manen, 1990). The phenomenological gaze (Van Manen, 1990) was focussed on gaining an understanding of the everyday experience of recovery from ankle fracture. There was an awareness of the social, cultural and historical context of the person, and temporality as the past, present and future is considered part of being human within their lifeworld (Heidegger, 1962). Prior research identified embodied endurance during the early phase of recovery (Keene et al., 2016). This study aimed to extend understanding and knowledge by exploring the experience of everydayness 6 months after injury.

The method was unstructured interviews to elicit participants’ lived experience of recovery from ankle fracture. The lead question was, what has it been like for you since you fractured your ankle? This was supported by prompts such as tell me more about that, how did you feel, what did you think, what helped or hindered at that point? Participants had at least 24 hours to consider the study information and their participation and provide written consent. Interviews took place face-to-face in a private area of a hospital or a meeting room in a hotel. One participant had their partner with them during the interview. One interview took place via the telephone and therefore lacked the benefits gained from personal contact and responses to non-verbal interactions. Despite this, a meaningful conversation was held, including experience of the emotional impact of recovery when living alone. The interview was therefore analysed in the same way as the face-to-face interviews. Ethical permission was granted from the National Research Ethics Service Hampshire B Research Ethics Committee (18/SC/0281) 2^nd^ July 2018, and the trial registration number was ISRCTN16612336.

A purposive sample of 20 participants were interviewed to obtain a range of sex, age and treatment. The participants were aged 50 years and over (51–82, median 66, 12 females) and received surgical or non-surgical treatment (7 internal fixation surgery, 7 close contact casting, 6 walking boot), and all were immobilised for at least 4 weeks. They were 6 months after injury and from three NHS Foundation Hospital Trusts in the South of England, UK. The malleolar segment facture classification (Meinberg et al., 2018) for the participants is presented in Table 1.

**Table 1.** Malleolar Segment Fracture Classification for the Participants.

Fracture classification	Participant number
44A1, Tibia/fibula, malleolar segment, infrasyndesmotic, isolated fibular injury	P7
44A3, Tibia/fibula, malleolar segment, infrasyndesmotic fibular injury with a posteromedial fracture	P10
44B1, Tibia/fibula, malleolar segment, transsyndesmotic isolated fibular fracture	P2, P6, P8, P9, P11
44B2, Tibia/fibula, malleolar segment, transsyndesmotic fibular fracture with a medial injury	P1, P3, P4, P5, P13, P15, P18, P19, P20
44B3, Tibia/fibula, malleolar segment, transsyndesmotic fibular fracture with a medial injury and fracture of the posterolateral rim (Volkmann’s fragment)	P14
44C2, Tibia/fibula, malleolar segment, suprasyndesmotic, wedge or multifragmentary diaphyseal fibula fracture	P12
44C3, Tibia/fibula, malleolar segment, suprasyndesmotic, proximal fibular injury	P17
Missing data	P16

Interviews (20) were undertaken, 18 by ET and 2 by DJK, and in 6, both researchers were present. The interviews took place from May 2019 to January 2020 and were of 24–75 min duration (median 50 min). All the interviews were recorded and transcribed verbatim. One potential participant declined to take part. Analysis developed through listening, reading and writing to gain an understanding of each participant’s world (Van Manen, 1990). Units of meaning identified within sentences were coded, and codes with similar meanings were grouped together into categories. Those that contained several meanings were placed where they would best enhance understanding of the developing categories and themes. Categories were drawn together into themes, that demonstrate the structure of experience (Van Manen, 1990). Similarities and differences within codes and categories and across participants in relation to the findings as a whole were noted. There were many overlaps between the themes such as fear of not returning to normal, falling and further damage to their ankle. These were highlighted in themes where they were most relevant. Interpretation of meaning was led by ET with regular reflective discussions with DJK and the co-authors. The lead researchers were experienced researchers with prior experience of investigating recovery from ankle fracture, one was also a clinical practitioner. Patient and public involvement partners helped shape the study design, interventions, analysis, management and dissemination of the study. One PPI member has been working with the team throughout the investigations into treatment and recovery from ankle fracture and is co-author on this paper. The impetus to investigate ankle fracture, study design and set-up were supported by the Oxford-led UK Musculoskeletal Trauma PPI group. Discussions about the experience of ankle fracture, which aided the researchers’ reflective process during analysis, were held with 4 PPI partners who met 4 times. An in-depth understanding of experience within the categories and themes occurred after 18 interviews, and 2 more interviews were untaken to reflect on the decision to stop interviewing.

Rigour drew on the concept of trustworthiness (Lincoln & Guba, 1985). The lead researchers (ET and DJK) were immersed in the data, and analysis of individual experience was drawn together into a representation of the experience of the group. Researchers’ assumptions during interpretation were challenged through reflection. Information about relevant literature, the sample, study design and findings were provided to aid a review of the transferability of the findings to other populations.

## Findings

### Getting on With Daily Life

Getting on with life after ankle injury was the lived experience of accepting what it feels like to be vulnerable and needing to be safe, while moving forward and hoping their abilities will improve. This was enacted through the following themes: i) ‘being vulnerable’ with categories of ‘being injured’ and ‘being able to walk again’, ii) ‘being safe’ with categories of ‘being fragile’ and ‘being careful’ and iii) ‘being myself’ with categories of ‘being determined’ and ‘being challenged’. The categories and themes with examples of participants’ quotes are presented in Table 2.

**Table 2.** Themes, Categories and Examples of Participants’ Quotes With the Code in Brackets.

Theme	Category	Examples of participants’ quotes with the code in brackets
‘Being vulnerable’ was participants’ experience of biopsychosocial disruption to daily life, where they were dependent on others, struggled to walk and feel confident and were fearful of falling and causing further damage to their ankle.	‘Being injured’ conveyed the disruption to daily life and dependency on others that impacts on wellbeing.	You don’t realise that trauma affects your wellbeing or anything, even the few days after it all happened, I said I’m fine but you’re not. Little things had happened to you and your body goes into shock really. P12 female, 44C2 (the unknown) I think the word is vulnerable, maybe I feel a bit more vulnerable because you never think this is going to happen. P3 female, 44B2 (being vulnerable) Well that’s it yes and I’ve never come across it in life before. I’ve been fit and active all my life and stuck there with my leg stuck in the air for two months is not my cup of tea. P11 male, 44B1 (being stuck) Well I always said, what’s a year out of 76 if I get better, but then some days I thought, I’m never going to walk again and that’s a bit low making, and I’m still limping. P14 female, 44B3 (being low)
	‘Being able to walk again’ was a struggle and participants lacked confidence. They worried about falling and causing further damage to their ankle.	It was once the plaster came off and then it got tricky again, and that’s another whole story. I went back to square one and had to work forward again. With the plaster on it didn’t hurt and I could walk around quite well at the start, with a bit of support, mostly with this trolley with wheels. P8 female, 44B1 (back to square one) You can’t really still walk then, you can’t just put your shoes on and off we go. You’re still in a wheelchair or hobbling on a pair of crutches, and it takes you a bit of time to get a bit of courage to actually put your weight on it [your foot], but of course the physio teaches you to do that. I don’t think unless you saw a physio at that stage, you wouldn’t actually stand on both legs. P17 female, 44C3 (courage to weight bear) I think it’s a matter of confidence, isn’t it, this walking lark because I was frightened of falling because when I was in the trauma unit here I had the operation, and the next day they gave me crutches, and I fell. P14 female, 44B3 (fear of falling) A few days had gone on and I can do it, but I was frightened to go out anywhere without the boot in case I did any damage. Until the doctor said the x-rays show that your bones have healed up, you don’t need the boot anymore. P12 female, 44C2 (fear of damage)
‘Being safe’ developed from participants’ awareness of their body as fragile, a sense of being less robust or damaged, and their desire to conserve and sustain the integrity of their body.	‘Being fragile’ occurred where the memory of the event, sensations, pain and stiffness acted as bodily reminders of injury and generated a desire to protect their body.	If I’m standing outside a neighbour’s house and haven’t got a handrail, and I’m on the steps, I’m really careful not to step back or move. So I’m definitely more aware that it was a stupid thing I did when I did it. P3 female, 44B2 (heightened awareness) I feel that I haven’t got quite the same control in the ankle on the injured foot as I had before but not to a major extent, it just doesn’t feel quite right. P18 male, 44B2 (lacking control) Painful is the only way I can describe it. Once I’ve put my weight on both legs the ankle really hurts and I know I should have done exercises, and so that’s the way it complains. It reminds me I need to look after myself, but we don’t listen, do we? P7 male, 44A1 (pain reminds me) There’s always a little bit of residual stiffness or something, or lack of movement. P1 female, 44B2 (residual stiffness)
	‘Being careful’ was participants’ response to living with reduced functional ability combined with a heightened awareness of danger. They were more cautious and often avoided activities.	I just feel that I need to be that little bit more careful. P18 male, 44B2 (being careful) If I’m carrying something, I make sure I’ve got a hand on the rail and it does make you readjust how you do things, and so in that sense I’m more conscious of it I suppose, it’s a good thing to be quite honest. P13 female, 44B2 (conscious of risks) A bit more cautious really and thinking, what’s the point of taking the risk if I can avoid doing it, yes. P11 male, 44B1 (avoidance) Yes and certainly I do quite a few stretches now first thing in the morning and that’s the time when I tend to struggle, the first time I go down the stairs in the morning I’m quite often going sideways down the stairs because I don’t trust my ankle. P10 male, 44A3 (lack of trust)
‘Being myself’ was how participants moved forward to regain or retain prior activities that supported their self-identity and worked hard to move away from notions of old age, while being challenged by the use of mobility aids.	‘Being determined’ was to push away from a disrupted self-identity where they felt or were perceived as older. They became attuned to their body and its capacity to recover and worked with existing conditions.	I wanted to make sure that I could do it and I was determined not to be the little old lady with the leg that wouldn’t work now. I wanted to get back as much as I could to everything. P12 female 44C2 (being determined) Well it was hard going, it was a bit miserable. First of all you had to have a walking frame which I think reminds me of some very elderly people and I don’t consider myself one of those. P4 female, 44B2 (I’m not old) It doesn’t help that I’ve had a bit of arthritis in my ankle for years as well, just to make things even more trouble but I’m getting there slowly. P10 male, 44A3 (hindered by arthritis) Perhaps I’m trying to fend off old age and that’s probably it. I’m fairly fit, I get out quite a lot. I’ve got a garden and an allotment and everything. I want to be able to do that again, then I’ve got to get my leg going again and I can’t let it pull me back and so I’ve had to keep pushing it forward. P8 female, 44B1 (pushing forward)
	‘Being challenged’ was a continuous process of learning to be more mobile. Participants worked hard to learn new skills with mobility aids, support boots and build up pace, distance and stamina while enjoying life and hoping for future improvements.	I struggled to get more than a couple of hundred yards if I’m honest on the crutches. P5 male, 44B2 (struggling) Of course at my age this is just a point that I found quite difficult, was using crutches because I did find that quite difficult because of my balance. I think as you get older, this is just a point, I think people do lose their balance. P20 female, 44B2 (loss of balance) I’m trying to step out quite far but I haven’t got the oomph [energy], I’m still going very slowly. P14 female, 44B3 (slow pace) It was nice because the first month I could really see a massive improvement on a weekly basis and I’m not seeing those improvements now but l know I’m getting better, it’s just a matter of time. P10 male, 44A3 (getting better)

### Theme 1: Being Vulnerable

Being vulnerable was participants’ experience of biopsychosocial disruption to daily life, where they were dependent on others, struggled to walk and feel confident and were fearful of falling and causing further damage to their ankle. Participants’ feelings, thoughts and daily life were affected, conveyed through the categories of ‘being injured’ and ‘being able to walk again’.

Category (i): Being injured conveyed the disruption to daily life and dependency on others that impacts on wellbeing. Being injured was a devastating blow for participants that disrupted all aspects of their life. They rarely had prior experience of fracture, treatments or recovery. Not being able to walk and carry out normal daily activities and being dependent on others were shocking. They felt vulnerable and disabled. The long-term implications were impossible to imagine in the early phase of treatment and recovery, and realisation evolved over time.

Since my injury. I mean it has just completely wiped out my life. After breaking my ankle I had no idea how it was going to be. Participant 16 (female, missing data)

The inactivity, loneliness and dependency of the early phase of non-weight bearing were tedious and frustrating.

Yes I suppose it’s more psychological than physical because if I’d had to do it again. I don’t know whether I would cope to be honest. Participant 5 (male, 44B2)

Being dependent on family and friends for support was challenging. Participants stoically endured this period but found it stressful and felt powerless. It was a real struggle to live during the non-weight bearing period and homes were adapted.

I can’t even carry a cup of tea across the room and so I had chairs around the room so that I could go kneel and drink tea or eat my breakfast or something. Participant 13 (female, 44B2)

Feeling very low was a common experience in the early phase of injury, or participants felt they would feel depressed if they didn’t have support. Being stoic and just getting on with life helped to sustain mood over time. Being able to weight bear again was a relief, and the joy of being able to do basic daily activities while standing was profound.

Yes just being able to walk to the toilet. The first time I managed that I thought god this is wonderful, it’s a big thing in your life, all of a sudden you can walk to the toilet. Participant 3 (female, 44B2)

Category (ii): Being able to walk and weight bear was a struggle and participants lacked confidence. They worried about falling and causing further damage to their ankle. The transition to weight bearing had to be planned, was hard work, required determined effort and caused disruption to the flow of everyday life. It was a stark change in mental focus from a determined effort to ‘don’t stand on that leg’ (P17 female, 44C3). Support from physiotherapists and families was appreciated, and courage was required to weight bear.

I was still using a crutch right at the beginning and walking any distance was hard work, which was alien to me. Completely. Doing anything was really, really hard work, just because I think my ankle had hardly moved from the month it was in the boot. Then I came out of the boot and then it just felt awful. Literally just trying to balance on it for a couple of seconds was nigh on impossible. Participant 10 (male, 44A3)

Needing to trust the ankle was important to feeling confident in weight bearing. Participants could feel disassociated from their foot.

It sort of feels weird to start off with, it doesn’t quite feel like your foot because all the muscles are very weak and so on. Well it’s learning to trust your foot again really. Participant 13 (female, 44B2)

There was a struggle with a fear of falling and a lack of confidence in their ability to walk. Participants changed their behaviour and how they viewed surfaces and steps and planned where they would walk to avoid risk. Poor balance and prior experience of falling further reduced their confidence.

Going up and down these steps I’m so careful and I’m so worried I’m going to fall again and do the same thing all over again. Participant 16 (female, missing data)

Living through the early days of injury, adjusting to loss and being dependent while non-weight bearing were hard work. Getting back to walking was a real challenge. Participants struggled to put weight on their foot, felt they lacked muscle strength, balance and confidence and worried about falling. They moved forward by taking measures to protect themselves, by ‘being safe’.

### Theme 2: Being Safe

Being safe developed from participants’ awareness of their body as fragile, a sense of being less robust or damaged, and their desire to conserve and sustain the integrity of their body. Consequently, they had a heightened awareness of the need to protect themselves and avoid further injury. Being safe was conveyed through ‘being fragile’ and ‘being careful’.

Category (i): Being fragile occurred where the memory of the event, sensations, pain and stiffness acted as bodily reminders of injury and generated a desire to protect their body. The immense impact of injury contrasted with the minor nature of the event, such as falling off a curb. There was a strong desire to avoid it happening again driven by a vivid memory of the event combined with their experience of struggling to recover.

It’s just I don’t want to do it again basically…I am nervous about it to a certain degree. Participant 10 (male, 44A3)

Unusual sensations in the ankle acted as a reminder of the injury and increased their awareness of their injured ankle. The sensations themselves such as feeling odd, tight and like a rubber band were often not debilitating, and participants felt they were a ‘small price to pay’ (P5 male, 44B2).

The injury just feels horrible, it felt really jelly-like and unstable and vulnerable. Participant 1 (female, 44B2)

All participants had experience of pain, particularly early in their recovery. Some were surprised how little pain they had overall, and others described themselves as having a high pain threshold and tolerated pain to avoid taking medication. However, pain was a common experience particularly first thing in the morning, and sharp shooting pains that often occurred without warning were debilitating and could be ‘as painful as breaking’ the ankle (P8 female, 44B1). Pain was expressed in many ways from sharp shooting pain to clicks, aches and discomfort but also pins and needles and mini electric shocks. Resting the ankle and medication helped. Pain in other areas of their body could distract participants from their painful ankle but also the experience of pain could be unexpected.

Swelling was a visual reminder of injury and could be challenging; it changed the look of their leg and was hard to explain to others. Legs felt heavy, uncomfortable or like ‘your feet are in cement’ (P4 female, 44B2). It could reduce over time or be persistent. Participants actively managed their activities, time walking or sitting with their legs raised to contain the swelling.

The swelling isn’t painful, it’s just unsightly especially when I wear shorts…so yes wearing shorts, it’s ‘what’s wrong with your leg?’ Participant 5 (male, 44B2)

Stiffness in the ankle could ease with exercise. However, residual stiffness was considered normal.

Visual and sensory reminders of injury combined to exacerbate feelings of fragility. Participants worked around sensations of pain, swelling and stiffness to get on with daily life. However, they were shocked by the impact of injury on their body, watched for changes and were surprised by the unpredictability of recovery.

Category (ii): Being careful was participants’ response to living with reduced functional ability combined with a heightened awareness of danger. They were more cautious and often avoided activities. It involved thinking through and planning activities prior to undertaking them, scanning ahead and knowing what surfaces, inclines or distance were manageable and then taking measures to make things easier or to avoid activities. Heightened awareness of danger led participants to curtail their activities to fit with their functional ability and avoid taking unnecessary risks.

I had a feeling of not being able to run away which you take for granted as part of your feeling safe in the world. Participant 15 (male, 44B2)

When confronted with uneven or challenging surfaces and stairs, three sources of danger were lack of pace, balance and flexibility. Lack of pace was challenging, and extra time was needed to negotiate activities and be ‘that little more careful’ (P18 male, 44B2). Stairs were a major source of perceived danger. Building up confidence was required to overcome the fear of the stairs and memory of a fall on the stairs. Planning was required for stairs, and participants moved slowly, held onto handrails, came down sideways or were supported by family and friends.

I cannot come down, face down forward. I come down the stairs one foot sideways. In the morning I’ve got the bannister one side and the wall the other side and so I always come down with the wall behind my back and so I’m pushing against the wall. Coming down the stairs, side step all the way down the stairs. Participant 12 (female, 44C2)

Walking on uneven surfaces with confidence was challenging due to lack of flexibility and balance. Building up their ability to feel steady on uneven surfaces helped some to return to work or recreational walking, and others avoided them. None of the participants were walking on uneven surfaces to the same degree as they did prior to injury.

Sometimes, you just don’t feel steady on your feet over uneven ground as I did beforehand**,** whether that’s psychological or not wanting to put all my weight on one leg in a rut or whatever and so I try and avoid things like that. Participant 5 (male, 44B2)

Participants felt they were recovered in many ways, but there still remained a sense of apprehension and the need to be careful.

Being careful was a way to stay safe and minimise risk. Many participants slowly improved their pace, balance and flexibility over time, but the need to be careful was still evident at 6 months after injury.

### Theme 3: Being Myself

Being myself was how participants moved forward to regain or retain prior activities that supported their self-identity and worked hard to move away from notions of old age, while being challenged by the use of mobility aids. Participants worked with their body to increase mobility, regain their life and aimed to get back to being normal. This was enacted through ‘being determined’ and ‘being challenged’.

Category (i): Being determined was to push away from a disrupted self-identity where they felt or were perceived as older. They became attuned to their body and its capacity to recover and worked with existing conditions. Participants often felt older and disabled and felt that others perceived them in the same way. This constituted a loss of self-identity. This was stoically tolerated when non-weight bearing but rejected once weight bearing. Participants were often disappointed by their lack of ability.

My movements are still impaired in ways that it made me feel old… I was starting to get some aches and pains but now some of the things I do I feel so embarrassed because I feel like how an old person looks. Participant 16 (female, missing data)

Old age was rejected by returning all walking equipment as soon as possible. However, there was a heightened awareness of ageing, ‘it’s all going to be downhill’ (P6, female, 44B1), the slow pace of their recovery and the way their limitations mirrored those associated with being older. Accepting limitations and developing self-comforting strategies could help support participants’ determination to keep active.

Deliberately walking slowly, looking at the view, so that no one thinks you might be struggling. You’ve got to put a brave face on it and pretend you’re going slowly. That was my mind set and if people thought you were struggling, just to go slowly and serenely, and let them go past. Participant 8 (female, 44B1)

Being determined to move forward, participants became attuned to their body and its capacity to recover. There was an awareness of limitations and a need to plan normally taken-for-granted activities and pace activities to avoid payback (if they did too much) in the form of pain, stiffness or tiredness. Participants became accustomed to the way their body works and attuned to the degree of exercise that they could manage, balancing activity and rest, pacing their activity and learning what exercise helped recovery.

It was like your body was telling you that you’d done too much at that point and there was a little bit of anxiety about had I done too much, had I put it back somehow, my recovery or whatever. Participant 13 (female, 44B2)

Participants could feel a dissonance between their mind and body as they struggled to relearn prior taken-for-granted activities, such as dancing where ‘your mind wants to do one thing and your body won’t go that way’ (P6, female, 44B1). Being determined also encompassed working within existing mobility limitations from chronic conditions or concerns about a new diagnosis, for example, osteoporosis, which could affect their confidence to move forward.

It’s probably sort of 30% caution about my ankle and 70% the fact that I don’t like doing it anyway, because I get a bad back and so I don’t really think the ankle injury is causing a great impediment. Participant 11 (male, 44B1)

Being determined conveyed a desire to avoid being seen as older or disabled. Participants sustained their progress forward by becoming attuned to their body, learning how far they can push them without payback and worked around existing conditions.

Category (ii): Being challenged was a continuous process of learning to be more mobile. Participants worked hard to learn new skills with mobility aids, support boots and build up pace, distance and stamina while enjoying life and hoping for future improvements. Learning to use mobility aids was challenging, and few participants achieved mastery. Walking frames were considered sturdier but led to painful shoulders and hands. Crutches were often discarded, moving the crutch was difficult and a good pace and distance rarely achieved. The use of crutches on stairs created anxiety; few people felt confident and felt they needed ‘somebody to really teach you to give you the confidence’ (P17 female, 44C3).

Walking boots were either valued or disliked. The boots could provide a sense of safety and protection from damage but also impeded walking, created an uneven gait and were heavy, cumbersome and disabling. Walking proficiently was a gradual staged process.

For a long while I’d lost the impetus to walk and so it had to be slow, it had to be quite relaxed, as if I’m deliberately walking slowly and not rushing anywhere. Now I can get a bit of steam up and so I have got the impetus back when I walk and if I choose to walk a little bit quicker then I can do it, and vary the pace. Participant 8 (female, 44B1)

Being challenged was a continuous incremental process of sustaining activity. Those that had physiotherapy identified their pleasure with their degree of progression and valued therapist support for choosing the exercises and providing encouragement. Gradually over time, activities that were highly challenging became taken for granted and activities progressed to new challenges. Participants enjoyed life and felt they were getting on and doing the things that were important to them. However, on further exploration, all had latent potential, activities such as squatting, kneeling, running and mountain walking that they had accepted they would not do again or were hoping they would be able to do in the future. A few were actively seeking help to progress their abilities. Participants hoped to be better but felt older and wondered if they would ever be the same as they were pre-injury.

I’ve got to be cheerful, but just didn’t appreciate how long it was going to take. It’s December now and I did it in February and no way I’m 100% now. Its months and months and months away, ten months away. Participant 16 (female, missing data)

## Discussion

The findings show how ‘getting on with daily life’ provides the momentum for recovery. Being injured changed the participants’ experience of the world, and feelings of being vulnerable, being safe and being myself became paramount as they worked their body in order to regain past abilities. At 6 months after injury, participants expressed their experience as: (i) feeling vulnerable as a result of injury, their experience of not being able to walk and dependency on others; (ii) needing to feel safe while living with constant reminders of fragility and being careful in their approach to activity and risk and (iii) working hard to sustain their sense of self, moving away from feeling older and disabled, and working to become increasingly mobile while aware of their lost function.

The implications for treatment and rehabilitation are that support maybe required for: (i) mental wellbeing throughout periods of increased vulnerability that include consideration of self-identity, ageing and disability, and counselling on prognosis, (ii) developing confidence and self-efficacy in decision making around being safe and (iii) training where further functional gain could enhance quality of life. A move towards treatments that facilitate earlier weight bearing (Bretherton et al., 2021) or maintenance of weight bearing status may reduce the detrimental effects of dependency and loss of mobility. The health benefits of maximising recovery in this age group have potential to mitigate the impact of increasing frailty with age and effect of co-morbidities on overall health. Lack of balance and reduced walking speed, identified in this study, can be indicators of frailty (Clegg et al., 2013). Injury in this population can also have a deleterious effect on quality of life (Van Son et al., 2016). Ankle fracture in this study was a point of transition where elements of being vulnerable, being safe and being myself resonated with concepts within ageing and chronic health.

### Being Vulnerable

This study adds to prior definitions of vulnerability in recovery from lower limb injury (Keene et al., 2016; Tutton et al., 2018). It broadens the definition to include recovery through limited weight bearing, early ambulation and up to 6 months. In early recovery, vulnerability was evident in the emotional response to injury, loss of function, social life and dependency on others. Enduring dependency when non-weight bearing, intense interdependent relationships and finding creative ways to live supports prior studies of ankle injury (Keene et al., 2016; McKeown et al., 2020). The transition to weight bearing was often disappointing, though longed for and viewed with relief; it was the beginning of their recovery rather than the end of their problems. Retraining their body to put weight through their foot was hard work and they struggled with many symptoms, evident in other studies of ankle injury (Jensen et al., 2021; Keene et al., 2016; McKeown et al., 2020; McPhail et al., 2012). Worries about causing further damage is also identified after major injury (Costa et al., 2018; Ekegren et al., 2020) and often includes fear of falling (Keene et al., 2016; McKeown et al., 2020). Despite the benefits, weight bearing without a cast or boot was often a setback due to the slow pace of walking, and the need to rethink and renegotiate all aspects of daily life. Inner resources of courage and confidence to trust their ankle and enact their plans were important. Fear of falling, for older people in the community, can link to increased frailty (de Souza et al., 2022). Confidence in their ability can diminish, particularly with the experience of a fall, and they can struggle to keep going (Underwood et al., 2021)*.* Fear avoidance beliefs, as found in this study, are also evident in older populations and can be linked to greater levels of disability (Camacho-Soto et al., 2012). Getting on with daily life in our study provided clear direction for overcoming vulnerability but low mood could hamper progression. As in frail older people (Underwood et al., 2021), social connectedness in the context of vulnerability could support confidence for progression. Understanding ongoing vulnerability and the impact of having been dependent may enable families/friends and professionals to support confidence-building activities within the context of daily life.

### Being Safe

Being safe reflected participant’s awareness of their body as fragile and the need to protect their body from further damage. Injury changed their experience of their body, how it looked and felt and what they could safely do, similar to that identified in recovery from open fracture of the lower limb (Rees et al., 2019). To maintain a sense of being normal, participants could dissociate from their broken ankle as in other studies of injury (Costa et al., 2018; Keene et al., 2016). Continuing sensations such as pain can be an ongoing problem in ankle injury (Jensen et al., 2021; Keene et al., 2016; McKeown et al., 2020; McPhail et al., 2012). Healthcare professionals can underestimate pain (Seers et al., 2018), and neuropathic pain in lower limb injury can be associated with poorer recovery (Keene et al., 2021). Balancing the degree of pain by reducing or avoiding activities was similar to integrating chronic pain into daily life, where the body can feel like it is working against the person (Toye et al., 2021). In this study, the need to be safe while maximising daily activity led to ‘being careful’ as a way of living with injury. Participants kept active while being vigilant and planning events, routes and timing of activities to ensure safe negotiation of space and avoidance of unsafe spaces. Being careful is evident in studies of ageing (Gardiner et al., 2017) and the experience of people living with osteoporosis (Hamed et al., 2021). A similar concept ‘constant vigilance’ is identified in recovery from ankle replacement or fusion for osteoarthritis (Pinsker et al., 2020). Awareness of the body as failing, pain and sensations heightened vigilance, and lack of trust in the body suggests supportive strategies that increased perceptions of safety may be needed to enhance integration of ankle injury into daily life.

### Being Myself

This study identified the impact of injury on participant’s experience of being themselves and their self-identify. Participants felt the sudden change of looking and feeling older and experienced loss of pace, speed, agility, spontaneity and freedom to move, elements indicative of youth and vitality. They did not feel ready for old age or for feelings reflected in the oldest old of being ‘diminished’, with a body in decline and a reduced contribution to the world (Toye et al., 2020). Participants strove to integrate their ankle injury into daily life reflective of a determination to age well or harmonious ageing, unlike successful ageing with its connotations of absence of disease (Moore et al., 2014)*.* There was a resonance with the concept of ‘preserving self’ (Vann-Ward et al., 2017), a drive to maintain as much of their youthful ‘self’ as possible. In striving to regain their identity, they trained their body and found walking aids and managing existing co-morbidities challenging, as in other studies of injury (Keene et al., 2016; McKeown et al., 2020)*.* In moving forward, there was acceptance that pre-injury activities could not be undertaken, they were adapted or they were avoided. Acceptance may have a role in the experience of quality of life and mental health (Aaby et al., 2020) and in older people living with chronic pain (Moore et al., 2014). In this study, participants felt grateful and lucky they had a future and were moving forward, also noted in living with chronic pain (Toye et al., 2021). However, they were concerned about the activities they were still unable to do, a loss of activity that may lead to an increase in frailty (Clegg et al., 2013) affecting participant’s ability to lead a fulfilled life.

### Limitations

A limitation of the study sample was that it did not include ethnic diversity. It also did not include people with cognitive impairment due to the interactive nature of the intervention. Further information about the context and socioeconomic nature of sample may have aided transferability to other populations and contexts. In addition, the sample included those who required definitive management with surgery, or non-surgical management of ankle immobilisation for at least 4 weeks, so may not have included those with less severe ankle fractures.

## Conclusion

This study identifies the lived experience of ankle injury, the work required to regain prior skills but also their continued loss of ability. Despite participants’ hope to return to normal, often the route to achieve this was not evident. Activities were constrained by functional ability but also a need to feel safe and manage the risk of reoccurrence. Understanding the experience of vulnerability, safety and self-identity may provide the basis for developing interventions that support recovery and maximise potential for vitality and ageing well. Ankle injury is a point of transition where early help with self-management of injury may reap benefits further along the ageing continuum.
